# Sex-specific differences in intestinal microbiota associated with cardiovascular diseases

**DOI:** 10.1186/s13293-024-00582-7

**Published:** 2024-01-19

**Authors:** Helena Garcia-Fernandez, Antonio P. Arenas-de Larriva, Javier Lopez-Moreno, Francisco M. Gutierrez-Mariscal, Juan L. Romero-Cabrera, Helena Molina-Abril, Jose D. Torres-Peña, Diego Rodriguez-Cano, Maria M. Malagon, Jose M. Ordovas, Javier Delgado-Lista, Pablo Perez-Martinez, Jose Lopez-Miranda, Antonio Camargo

**Affiliations:** 1 Lipids and Atherosclerosis Unit, Department of Internal Medicine, Hospital Universitario Reina Sofía, Cordoba, Spain; 2 Department of Medical and Surgical Sciences, Universidad de Cordoba, Cordoba, Spain; 3 Maimonides Institute for Biomedical Research in Cordoba (IMIBIC), Cordoba, Spain; 4 CIBER Fisiopatologia Obesidad y Nutricion (CIBEROBN), Instituto de Salud Carlos III, Madrid, Spain; 5 Department of Applied Mathematics I, University of Seville, Seville, Spain; 6 Clinical Analysis Service, Reina Sofia University Hospital, Cordoba, Spain; 7 Department of Cell Biology, Physiology, and Immunology, University of Cordoba, Cordoba, Spain; 8 Nutrition and Genomics Laboratory, Jean Mayer USDA Human Nutrition Research Center on Aging, Tufts University, Boston, MA USA; 9 IMDEA Food Institute, Madrid, Spain

**Keywords:** Gut microbiota, Dysbiosis, Sexual dimorphism, Cardiovascular diseases, CORDIOPREV

## Abstract

**Background:**

Cardiovascular diseases (CVD), including coronary heart disease (CHD), display a higher prevalence in men than women. This study aims to evaluate the variations in the intestinal microbiota between men and women afflicted with CHD and delineate these against a non-CVD control group for each sex.

**Methods:**

Our research was conducted in the framework of the CORDIOPREV study, a clinical trial which involved 837 men and 165 women with CHD. We contrasted our findings with a reference group of 375 individuals (270 men, 105 women) without CVD. The intestinal microbiota was examined through 16S metagenomics on the Illumina MiSeq platform and the data processed with Quiime2 software.

**Results:**

Our results showed a sex-specific variation (beta diversity) in the intestinal microbiota, while alpha-biodiversity remained consistent across both sexes. Linear discriminant analysis effect size (LEfSe) analysis revealed sex-centric alterations in the intestinal microbiota linked to CVD. Moreover, using random forest (RF) methodology, we identified seven bacterial taxa—g_UBA1819 (Ruminococcaceae), g_Bilophila, g_Subdoligranulum, g_Phascolarctobacterium, f_Barnesiellaceae, g_Ruminococcus, and an unknown genus from the Ruminococcaceae family (Ruminococcaceae incertae sedis)—as key discriminators between men and women diagnosed with CHD. The same taxa also emerged as critical discriminators between CHD-afflicted and non-CVD individuals, when analyzed separately by sex.

**Conclusion:**

Our findings suggest a sex-specific dysbiosis in the intestinal microbiota linked to CHD, potentially contributing to the sex disparity observed in CVD incidence.

***Trial registration*** Clinical Trials.gov.Identifier NCT00924937.

**Graphical Abstract:**

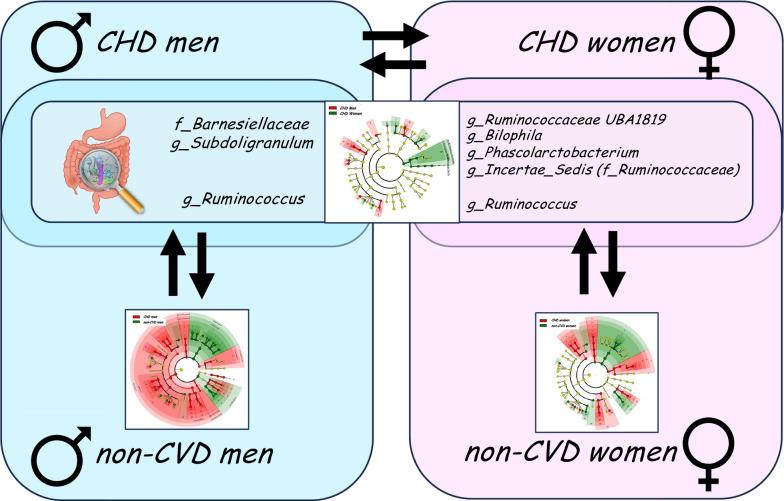

**Supplementary Information:**

The online version contains supplementary material available at 10.1186/s13293-024-00582-7.

## Highlights

- The incidence of cardiovascular disease is influenced by sex and appears with greater frequency in men than women.
- We hypothesized that the alterations in the intestinal microbiota associated with cardiovascular disease are different in men and women, which could influence the processes that interconnect intestinal microbiota and cardiovascular disease.
- Our results suggest that the dysbiosis of the intestinal microbiota associated with coronary heart disease seems to be partially sex-specific.
- The identification of the mechanisms responsible for sexual dimorphism in the incidence of metabolic and cardiovascular disease is of particular importance when developing effective strategies and therapies aimed at reducing their incidence and recurrence, which should be sex-specific.

**Supplementary Information:**

The online version contains supplementary material available at 10.1186/s13293-024-00582-7.

## Plain language summary

The frequency with which cardiovascular diseases occur differs in men and women as it appears with greater frequency in men. Moreover, it has been known for years that the community of bacteria living in our intestine, also known as the gut microbiota, influences the development of these diseases. Indeed, nowadays it known the influence of the intestinal microbiota in the development of atherosclerosis, the pathological process which is responsible for the three main causes of cardiovascular diseases: coronary heart disease, cerebrovascular disease and peripheral arterial disease. This study shows the differences in the community of bacteria living in the gut of men and those living in the gut of women, so that these differences could explain, at least in part, the differences in the frequency with which cardiovascular diseases appear between men and women. Our results suggest that the dysbiosis of the intestinal microbiota associated with CHD seems to be partially sex-specific, which may influence the sexual dimorphism in its incidence. Moreover, the identification of the mechanisms responsible for sexual dimorphism in the incidence of metabolic and cardiovascular disease is of particular importance when developing effective strategies and therapies aimed at reducing their incidence and recurrence. Indeed, the strategies and therapies used to treat the dysbiosis of the gut microbiota should be sex-specific.

**Supplementary Information:**

The online version contains supplementary material available at 10.1186/s13293-024-00582-7.

## Background

Cardiovascular disease (CVD) is a leading cause of mortality in western nations, with incidence rates displaying a notable sex-based dichotomy—men exhibit a higher prevalence compared to women [1]. In recent years, mounting evidence has underscored the role of the intestinal microbiota in the onset of atherosclerosis, the pathological underpinning of the three primary CVD manifestations: coronary heart disease (CHD), cerebrovascular disease, and peripheral arterial disease [2, 3].

The composition of the intestinal microbiota depends on the sex, in addition to other factors such as age, genetic background and the nutritional habits of the host organism [4–7]. In fact, intestinal microbiota transplant experiments in germ-free mice have demonstrated that the sex of the recipient animal shapes the composition of the intestinal microbiota [8].

Factors such as age, genetics, dietary habits, and notably, sex, significantly influence the composition of the intestinal microbiota [4–7]. Studies using germ-free mice have confirmed that the sex of the recipient animal contributes to shaping the gut microbiota [8]. Moreover, growing evidence has indicated that differences in intestinal microbial architecture between sexes may contribute to sex-based disparities in various pathological conditions, such as autoimmune and metabolic diseases [9–12]. This sex-specific influence is evident in our recent findings on the differential composition of gut microbiota in men and women with metabolic syndrome (MetS), which could partly explain the sex-based incidence of this syndrome [13].

In light of these insights, this study hypothesizes that the gut microbiota alterations associated with CVD differ between sexes, potentially influencing CVD-linked processes like endotoxemia, lipid and cholesterol metabolism, and the production of microbial-origin metabolites [2, 3]. Our goal is to examine the differential intestinal microbiota in men and women with CHD, within a population reflecting this disease's sexual dimorphism (CORDIOPREV study, 837 men and 165 women) [14]. We will compare these findings with a non-CVD reference population, living in the same geographical locale as the CORDIOPREV cohort, to account for shared confounding factors such as lifestyle, dietary habits, and genetic background.

## Methods

### Study population

The current work was conducted in framework of the CORDIOPREV study (Clinical Trials.gov.Identifier: NCT00924937, Registered 19 June 2009, https://classic.clinicaltrials.gov/ct2/show/NCT00924937), an ongoing prospective, randomized, open, controlled trial in 1002 patients with coronary heart disease (CHD) who had their last coronary event over six months before enrolling, and who followed two different dietary models (a low-fat diet and the Mediterranean diet) over a period of seven years in addition to conventional treatment for CHD [14]. CORDIOPREV inclusion and exclusion criteria can be summarized as follows: patients were eligible if they were over 20 years old but under 75, had established CHD without clinical events in the last 6 months, were thought to be capable of following a long-term dietary intervention, and did not have severe diseases, diseases of the digestive tract that involve episodes of diarrhea or an estimated life expectancy of less than seven years. Detailed inclusion and exclusion criteria have been previously described [15]. Fecal samples were available at baseline for a total of 726 patients from which 682 had not received treatment with antibiotics within 1 month before baseline sample collection. Moreover, 3 samples were discarded because low sequencing quality (N = 679).

As reference of non-CVD population, we used the cohort of 375 non-CVD individuals enrolled in the ONCOVER study as healthy controls (http://www.proyecto-oncover.es/), which represents a population without CVD recruited among the free-living population without oncological diseases or disabling diseases or whose severity implied a life expectancy of less than three years from the same geographical location as the population of CORDIOPREV study, who share many of the co-founding factors such as lifestyle, dietary habits and genetic background with the study population. Fecal samples were available for a total of 338 patients from which 329 had not received treatment with antibiotics within 1 month before baseline sample collection. Both studies, CORDIOPREV and ONCOVER, have been approved by the Reina Sofia (Cordoba) University Hospital Ethics and Research Committees. All the participants agreed to their inclusion in these studies. Their trial protocols and all the amendments were approved by the Reina Sofia University Hospital Clinical Research Ethics Committee, following the Helsinki Declaration and good clinical practice.

### Clinical plasma parameters

Blood was collected in tubes containing EDTA to give a final concentration of 0.1% EDTA. The plasma was separated from the red blood cells by centrifugation at 1500×*g* for 15 min at 4 ºC. Analytes in the frozen samples, blinded to the team members, were analyzed centrally by members of the laboratory research team at the Lipid and Atherosclerosis Unit at Reina Sofia University Hospital. The clinical plasma parameters were measured as previously described [6].

### Intestinal microbiota analysis

DNA extraction from feces was performed using the QIAamp DNAStool Mini Kit Handbook (QIAGEN, Hilden, Germany), following the manufacturer’s instructions. The intestinal microbiota was examined through 16S metagenomics on the Illumina MiSeq platform (Illumina, San Diego, CA, USA) and the data processed with Quiime2 software as previously described [16]. The sequences obtained in this study have been submitted to NCBI Sequence Read Archive (SRA) under the accession numbers PRJNA1000902 and PRJNA1000795. Further, sequencing data were analyzed and visualized using QIIME2 [17], using the DADA2 method [18]. We evaluated the bacterial alpha- [19] and beta-diversity [20], this latter analyzed by permutational multivariate analysis of variance (PERMANOVA). Taxonomy was assigned to the high-quality reads using q2‐feature‐classifier [21] with a sequence identity threshold of 99% interrogating the sequences with the SILVA database [22]. Linear discriminant analysis (LDA) effect size (LEfSe) was used to compare groups at baseline and visualize the results using taxonomic bar charts and cladograms [23]. In this analysis, to exclude bacterial taxa that were not present in the majority of samples, a cut-off for exclusion was fixed; only bacterial taxa containing sequence reads in at least 75% of total samples were considered. The Chi-square test was applied to establish differences in bacterial presence/absence in bacterial taxa containing sequence reads in at least 50% of the samples in at least one of the experimental groups. Multiple comparisons in the large-scale expression analyses were assessed by False Discovery Rate (FDR) using the Benjamini and Hochberg method. *P*-values < 0.05 and *Q*-values < 0.1 were considered statistically significant. To study the potential functionality of the gut microbiota, PICRUSt2 (Phylogenetic Investigation of Communities by Reconstruction of Unobserved States) analysis of 16S sequences was performed to predicate and identify differentially enriched pathways. Thus, PICRUSt2 [24] was used to impute MetaCyc pathway abundance from the original taxonomic assignment. Further, metabolic pathway data were compared by STAMP [25], a graphical software package that provides statistical hypothesis tests and exploratory plots for functional profiles. Data were compared by two-sided Welch’s *t*-test and filtered for false discoveries using the Benjamini–Hochberg method (*Q*-value filter > 0.1) and an effect size filter higher than 0.01.

### Statistical analysis

The statistical analysis of the data was carried out with SPSS statistical software (IBM SPSS Statistics version 28.0). One-way ANOVA was performed to calculate the statistical differences of the quantitative anthropometric and metabolic variables between groups. *P*-values < 0.05 were considered statistically significant. R software, version 4.2.3 (R Foundation for Statistical Computing, http://www.R-project.org/) for the random forest classifier carried out using the script of the caret package in R. To obtain more precise curves and assess the performance of the models, we used repeated tenfold cross-validation. The cross-validation error curves (average of ten validation sets each) and performance were averaged. The different taxonomic taxa were normalized by centering and scaling. The predictive value of each variable in the random forest models was calculated by Mean Decrease in Accuracy. The model’s performance was further evaluated through the AUC on the test set. pROC R package was used to calculate the confidence intervals for ROC curves.

## Results

### Baseline characteristics of the study participants

Based on sex (Table 1), we found CHD men to be younger compared to CHD women (*P* < 0.05). Interestingly, this sex-based differences in CHD patients were absent in non-CVD subjects. Conversely, higher triacylglycerides, along with increased fasting glucose and insulin levels, were observed in non-CVD men compared to non-CVD women (*P* < 0.05), a distinction absents among CHD patients. Men consistently demonstrated a higher waist circumference, blood pressure, and lower HDL-c than women across both CHD patients and non-CVD subjects. Differences in anthropometric and metabolic variables between CHD and non-CVD subjects are presented in Table 2.

**Table 1:** Baseline characteristic of the participants with available fecal samples in the study according to the sex

	CHD patients			Non-CVD subjects		
	Men	Women	*P*-value	Men	Women	*P*-value
*N* (men/women)	567	112	n.a	242	87	n.a
Age (years)	59.1 ± 0.4	62.8 ± 0.8	< 0.001	59.2 ± 0.6	59.6 ± 0.9	0.711
BMI (kg/m^2^)	31.1 ± 0.2	31.2 ± 0.5	0.821	29.5 ± 0.3	29.0 ± 0.6	0.393
WC (cm)	106.1 ± 0.5	99.0 ± 1.2	< 0.001	101.9 ± 0.7	93.6 ± 1.7	< 0.001
HDL-c (mg/dL)	41.2 ± 0.4	47.5 ± 1.2	< 0.001	43.7 ± 0.6	54.7 ± 1.3	< 0.001
LDL-c (mg/dL)	88.2 ± 1.1	91.7 ± 2.8	0.190	132.8 ± 2.0	130.9 ± 2.9	0.612
TAG (mg/dL)	136.5 ± 2.9	136.0 ± 7.3	0.953	122.1 ± 3.9	97.4 ± 4.6	0.001
Fasting glucose (mg/dL)	112.9 ± 1.6	115.8 ± 4.9	0.482	104.9 ± 1.4	93.8 ± 1.5	< 0.001
Fasting insulin (mU/l)	10.5 ± 0.4	10.8 ± 1.2	0.764	8.8 ± 0.4	6.7 ± 0.5	0.008
HbA1c (%)	6.63 ± 0.05	6.74 ± 0.13	0.409	5.75 ± 0.20	5.47 ± 0.07	0.408
Systolic BP (mmHg)	137.6 ± 0.8	141.7 ± 1.9	0.046	142.5 ± 1.2	136.9 ± 2.3	0.021
Diastolic BP (mmHg)	77.5 ± 0.5	74.2 ± 0.9	0.004	84.6 ± 0.7	78.5 ± 1.2	< 0.001

Values correspond to the mean ± SEM

*BMI* body mass index, *WC* waist circumference, *HDL-c* high density lipoprotein-cholesterol, *LDL-c* low density lipoprotein-cholesterol, *TAG* triacylglycerides, *BP* blood pressure

The statistical differences between groups were evaluated by one-way ANOVA

**Table 2:** Baseline characteristic of the participants in the study with available fecal samples

	CHD vs non-CVD in population			CHD vs non-CVD in men			CHD vs non-CVD in women		
	CHD	Non-CVD	*P*-value	CHD	Non-CVD	*P*-value	CHD	Non-CVD	*P*-value
*N* (men/women)	679 (567/112)	329 (242/87)	< 0.001	567	242	n.a	112	87	n.a
Age (years)	59.7 ± 0.4	59.3 ± 0.5	0.508	59.1 ± 0.4	59.2 ± 0.6	0.889	62.8 ± 0.8	59.6 ± 0.9	0.011
BMI (kg/m^2^)	31.1 ± 0.2	29.4 ± 0.3	< 0.001	31.1 ± 0.2	29.5 ± 0.3	< 0.001	31.2 ± 0.5	29.0 ± 0.6	0.006
Waist circumference (cm)	104.9 ± 0.4	99.8 ± 0.7	< 0.001	106.1 ± 0.5	101.9 ± 0.7	< 0.001	99.0 ± 1.2	93.6 ± 1.7	0.007
HDL-cholesterol (mg/dL)	42.3 ± 0.4	46.6 ± 0.6	< 0.001	41.2 ± 0.4	43.7 ± 0.6	0.001	47.5 ± 1.2	54.7 ± 1.3	< 0.001
LDL-cholesterol (mg/dL)	88.7 ± 1.0	132.3 ± 1.6	< 0.001	88.2 ± 1.1	132.8 ± 2.0	< 0.001	91.7 ± 2.8	130.9 ± 2.9	< 0.001
Triglycerides (mg/dL)	136.4 ± 2.7	115.5 ± 3.2	< 0.001	136.5 ± 2.9	122.1 ± 3.9	0.005	136.0 ± 7.3	97.4 ± 4.6	< 0.001
Fasting glucose (mg/dL)	113.4 ± 1.5	102.0 ± 1.1	< 0.001	112.9 ± 1.6	104.9 ± 1.4	0.002	115.8 ± 4.9	93.8 ± 1.5	< 0.001
Fasting insulin (mU/L)	10.5 ± 0.4	8.3 ± 0.4	< 0.001	10.5 ± 0.4	8.8 ± 0.4	0.015	10.8 ± 1.2	6.7 ± 0.5	0.003
HbA1c (%)	6.65 ± 0.05	5.67 ± 0.15	< 0.001	6.63 ± 0.05	5.75 ± 0.20	< 0.001	6.74 ± 0.13	5.47 ± 0.07	< 0.001
Systolic BP (mmHg)	138.3 ± 0.8	141.2 ± 1.0	0.029	137.6 ± 0.8	142.5 ± 1.2	0.001	141.7 ± 1.9	136.9 ± 2.3	0.119
Diastolic BP (mmHg)	76.9 ± 0.4	83.1 ± 0.6	< 0.001	77.5 ± 0.5	84.6 ± 0.7	< 0.001	74.2 ± 0.9	78.5 ± 1.2	0.005

Values correspond to the mean ± SEM

*BMI* body mass index, *HDL-c* high density lipoprotein-cholesterol, *LDL-c* low density lipoprotein-cholesterol, *TAG* triacylglycerides, *BP* blood pressure

The statistical differences between groups were evaluated by one-way ANOVA

### Diversity of the gut microbiota according to the sex

There were no significant differences in alpha diversity indices (Shannon, Simpson (1-D), observed features and Faith's phylogenetic diversity) between male and female CHD patients. However, both Shannon and Simpson alpha diversity indices were lower in CHD men compared to non-CVD men, and similarly lower in CHD women compared to non-CVD women (*P* = 0.007, *P* = 0.003, *P* = 0.004, and *P* = 0.005, respectively) (Table 3). In contrast, we found significant sex-based distinctions in beta-diversity among CHD patients, which held true across Jaccard and Bray–Curtis distances (qualitative and quantitative measures, respectively), as well as Unweighted and Weighted Unifrac distances (also qualitative and quantitative, considering bacterial phylogeny) (all, *P* < 0.05). Similar significant differences were found between CHD and non-CVD subjects, within each sex (Table 3).

**Table 3:** Alpha- and beta-diversity indexes

	CHD men vs CHD women	CHD men vs non-CVD men	CHD women vs non-CVD women	Non-CVD men vs non-CVD women
*N*	567/112 (men/women)	567/242 (CVD/non-CVD)	112/87 (CVD/non-CVD)	242/87 (men/women)
Alpha diversity				
Shannon	0.199	0.007 (lower in CVD)	0.004 (lower in CVD)	0.639
Simpson (1-D)	0.408	0.003 (lower in CVD)	0.005 (lower in CVD)	0.559
Observed_features	0.741	0.871	0.355	0.510
Faith-fd	0.612	0.386	0.421	0.107
Beta diversity				
Jaccard	0.001	0.001	0.001	0.001
Bray–Curtis	0.001	0.001	0.001	0.001
Unweighted_unifrac	0.001	0.001	0.001	0.001
Weighted_unifrac	0.001	0.001	0.002	0.002

*CHD* coronary heart disease, *CVD* cardiovascular disease, *Faith-fd* Faith’s phylogenetic diversity

### Differences in the gut microbiota composition according to the sex: LEfSe analysis

**Fig. 1:**
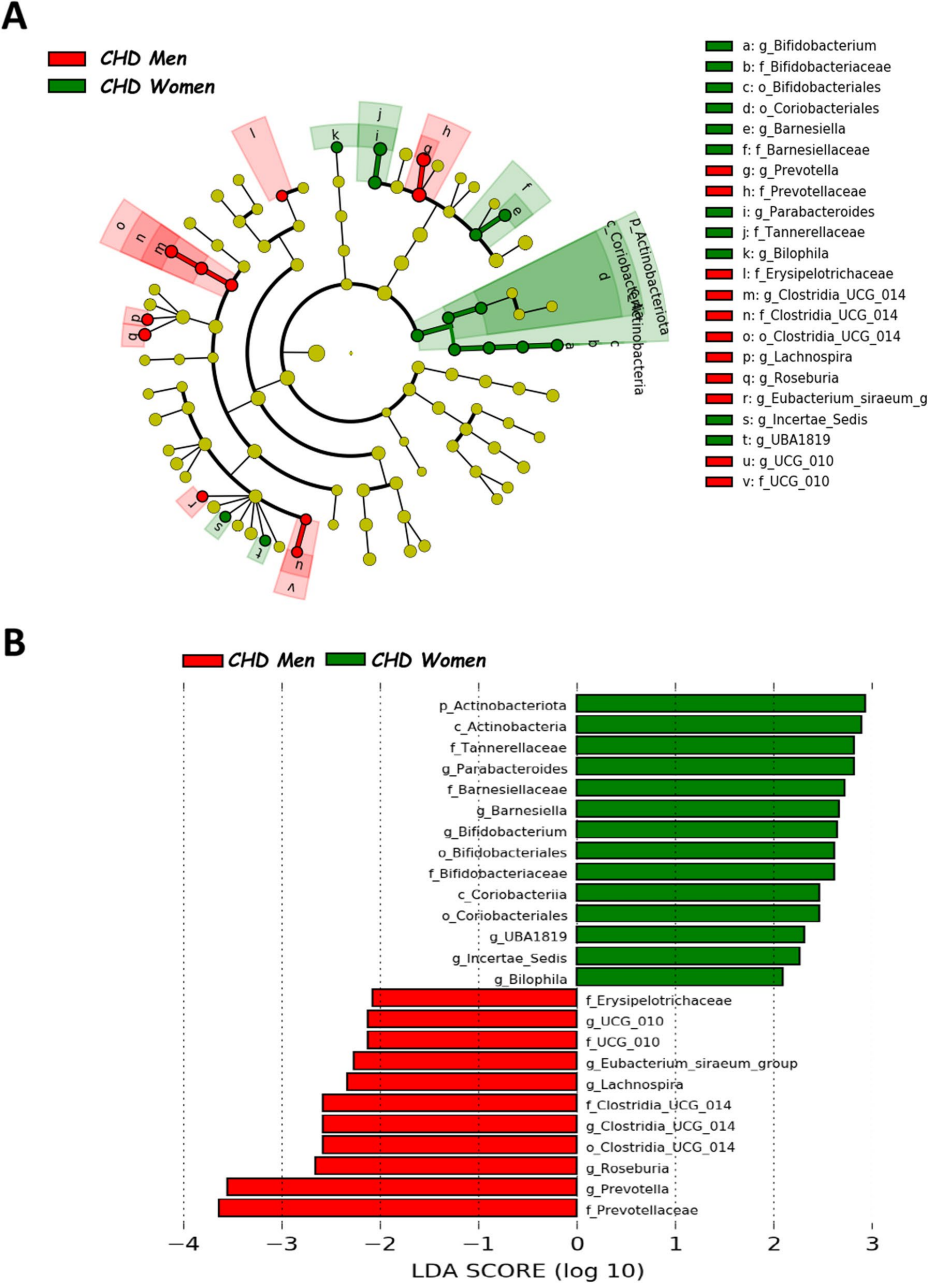
Differently abundant taxa in CHD patients according to the sex, based on LEfSe analysis. The colors represent the group in which the indicated taxa are more abundant compared to the other group. In a taxonomic cladogram, each successive circle represents a different phylogenetic level. **A** Cladogram: the order from the center to the outside is phylum, class, family and genus levels. Differing taxa are listed on the right side of the cladogram. **B** Linear discriminant analysis. The most differently abundant taxa between sexes are represented in a bar graph according to the LDA score (log 10), an estimation of the effect size. Only taxa meeting a *P* < 0.05 and LDA score significant threshold |> 2| are shown. g_UBA1819: g_UBA1819 (Ruminococcaceae); g_Incertae Sedis: Ruminococcaceae incertae sedis; f_UCG_010: f_UCG_010 (Oscillospirales). *CHD* coronary heart disease patients

**Fig. 2:**
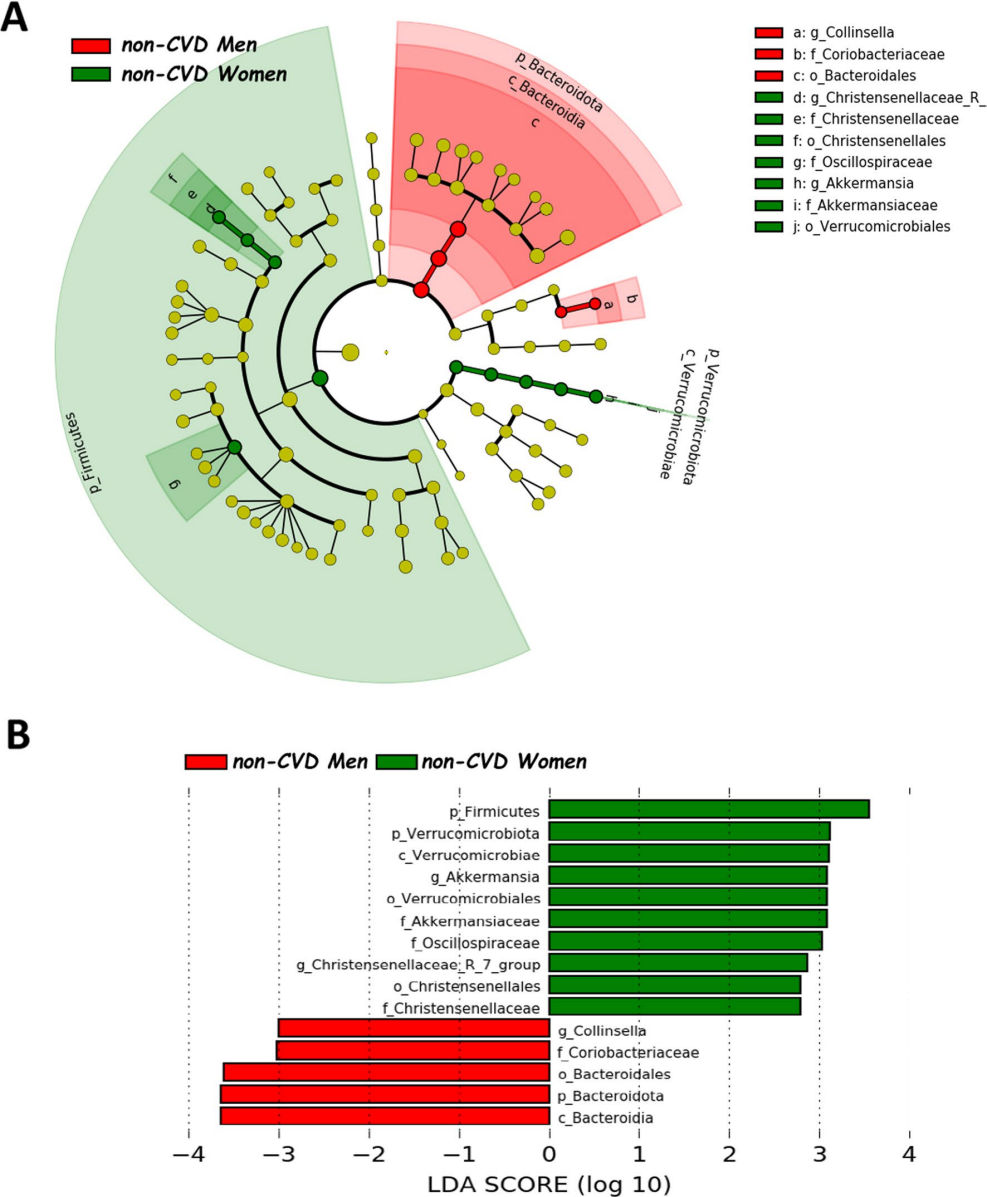
Differently abundant taxa in non-CVD patients according to the sex, based on LEfSe analysis. The colors represent the group in which the indicated taxa are more abundant compared to the other group. In a taxonomic cladogram, each successive circle represents a different phylogenetic level. **A** Cladogram: the order from the center to the outside is phylum, class, family and genus levels. Differing taxa are listed on the right side of the cladogram. **B** Linear discriminant analysis. The most differently abundant taxa between sexes are represented in a bar graph according to the LDA score (log 10), an estimation of the effect size. Only taxa meeting a *P* < 0.05 and LDA score significant threshold |> 2| are shown. g_UBA1819: g_UBA1819 (Ruminococcaceae); g_Incertae Sedis: Ruminococcaceae incertae sedis; f_UCG_010: f_UCG_010 (Oscillospirales). non-*CVD* non-cardiovascular disease individuals

**Fig. 3:**
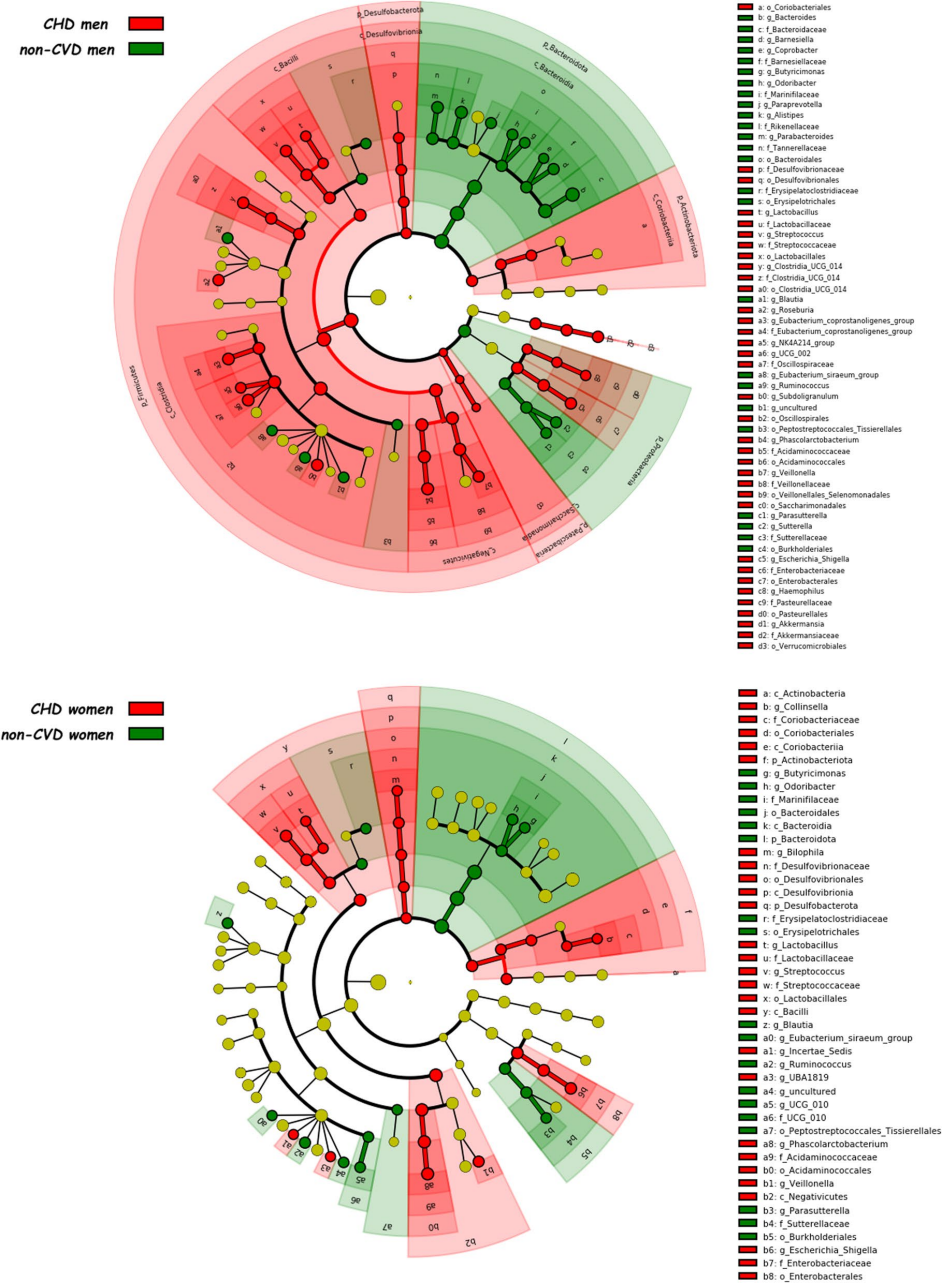
Differently abundant taxa between CHD patients and non-CVD subjects in men and women separately identified using LEfSe analysis. The colors represent the group in which the indicated taxon is more abundant compared to the other group. In a taxonomic cladogram, each successive circle represents a different phylogenetic level. The order from the center to the outside is phylum, class, family and genus levels. Differing taxa are listed on the right side of the cladogram. *CHD* coronary heart disease patients, *non-CVD* non-cardiovascular disease individuals

**Fig. 4:**
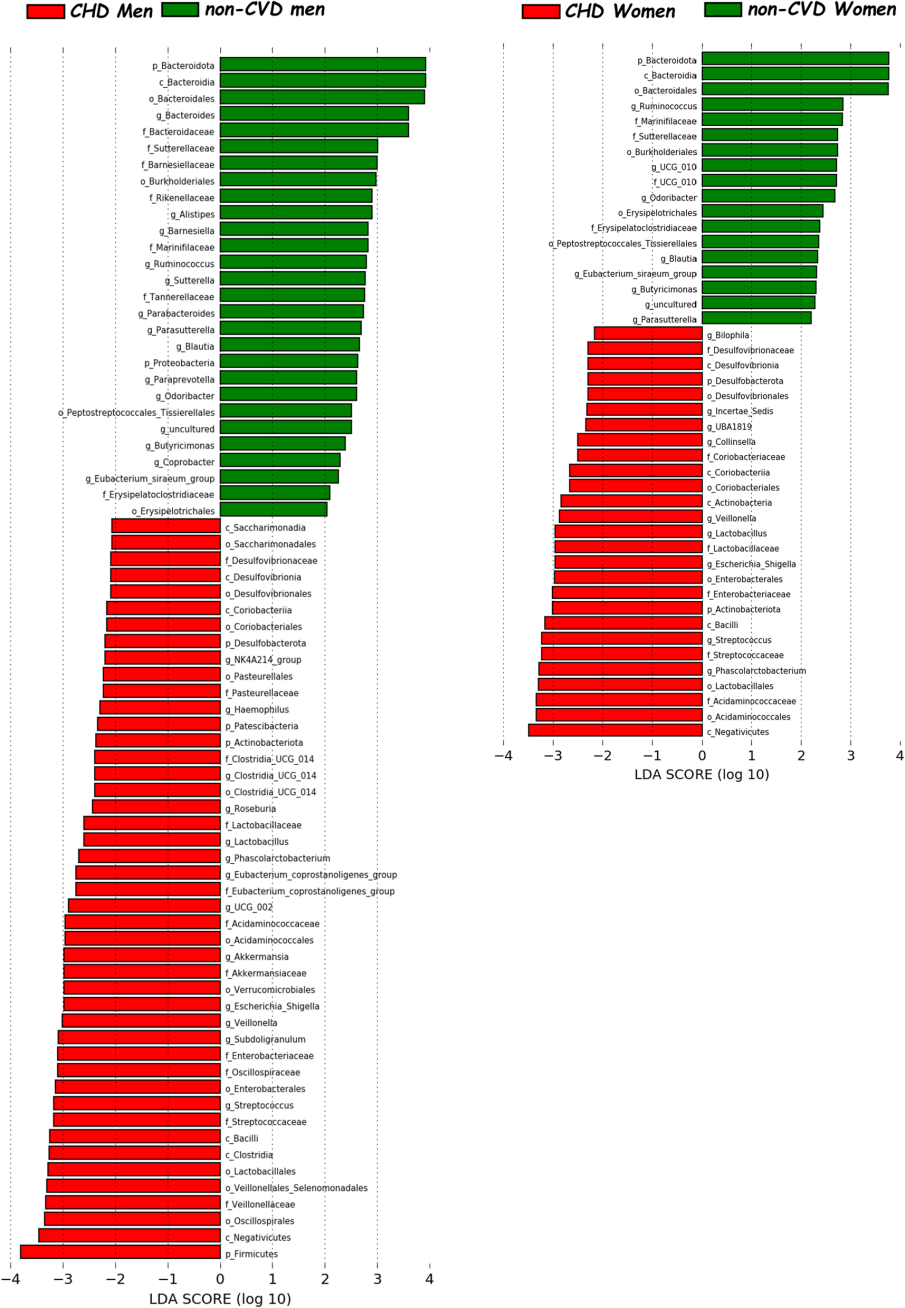
Linear discriminant analysis between CHD patients and non-CVD subjects in men and women separately. The most differently abundant taxa between sexes are represented in a bar graph according to the LDA score (log 10), an estimation of the effect size and in a taxonomic cladogram. Only taxa meeting a *P* < 0.05 and LDA score significant threshold |> 2| are shown. *CHD* coronary heart disease patients, *non-CVD* non-cardiovascular disease individuals

We employed LEfSe to identify taxonomic variations between the gut microbiota of male and female CHD patients. The intestinal microbiota of CHD men was characterized by a preponderance of *Clostridia*_*UCG*_*014* order (also *Clostridia*_*UCG*_*014* family and *Clostridia*_UCG_*014* genus), *UCG*_*010* family (also *UCG*_*010* genus), *Prevotellaceae* (also *Prevotella* genus) and *Erysipelotrichaceae* families, and *Eubacterium*_siraeum_group, *Lachnospira*, and *Roseburia* genera. By contrast, the gut microbiota of CHD women was characterized by a preponderance of *Actinobacteriota* phylum (also *Actinobacteria* class), *Coriobacteriia* class (also *Coriobacteriales* order), *Bifidobacteriales* order (also *Bifidobacteriaceae* family and *Bifidobacterium* genus), *Barnesiellaceae* (also *Barnesiella* genus) and *Tannerellaceae* families, and *Parabacteroides*, UBA1819 (Ruminococcaceae), Ruminococcaceae incertae sedis and *Bilophila* genera (Fig. 1). Moreover, we also analyzed the differences in taxonomic composition between non-CVD subjects according to the sex (Fig. 2). Additionally, we noted differences in taxonomic composition between CHD patients and non-CVD subjects, examined separately for each sex (Figs. 3, 4).

### Differences in the frequency of presence-absence according to the sex

**Fig. 5:**
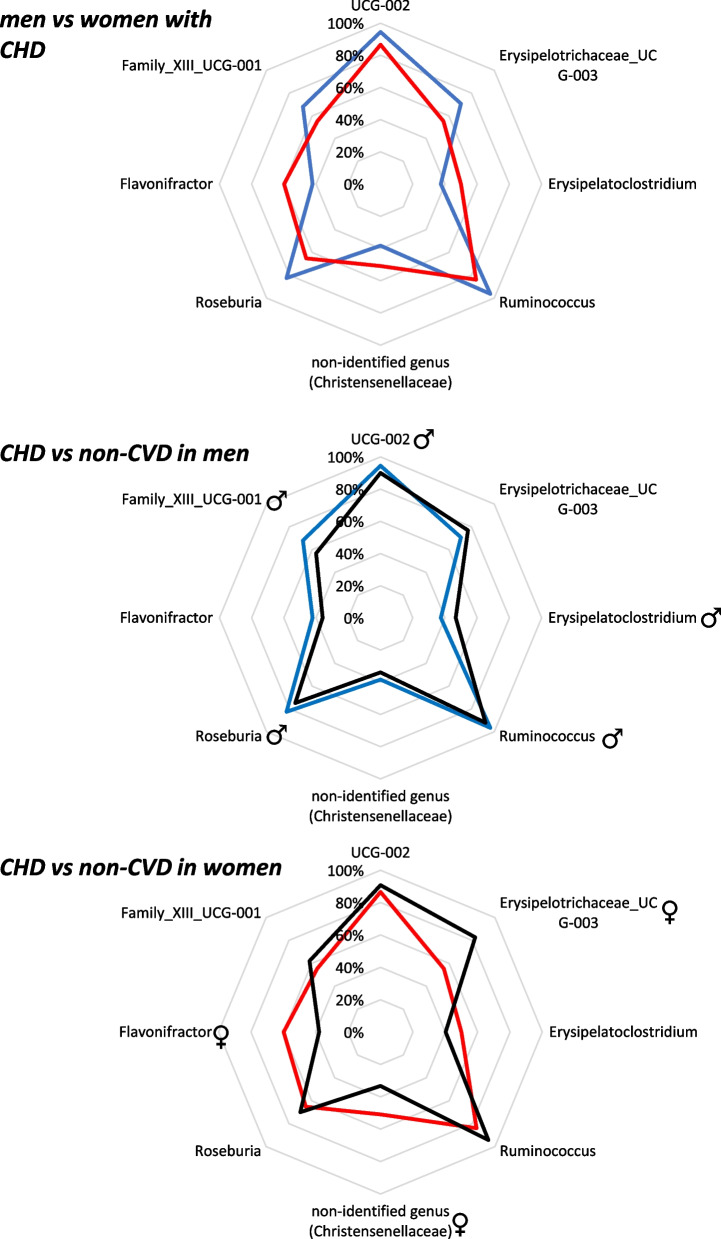
Differences in the frequency of presence-absence according to the sex: qualitative analysis. The Chi-square test was applied to establish differences in bacterial presence/absence at genus level

We examined the frequency bacterial genera between sexes and found significant differences in the occurrence of 14 bacterial genera between male and female CHD patients (*P* < 0.05 and *Q* < 0.1 in Chi-square test). In addition, frequency differences were observed for 35 bacterial genera between CHD men and non-CVD men, and 18 bacterial genera between CHD women and non-CVD women. Thirteen genera were distinct between CHD patients and non-CVD subjects for both sexes (Fig. 5).

### Identification of discriminant bacterial taxa according to the sex: data modeling

A random forest classifier was used to evaluate the different taxonomic taxa normalized on the datasets and their performance evaluated through the AUC of the 20 bacterial taxa most important identified by each model: (i) men and women with CHD and sex as factor (men vs women with CHD model: AUC of 73.0 (CI 67.8–78.2); (ii) men with CHD and men without CVD, and the presence of CVD as factor (CHD men vs non-CVD men model: AUC of 88.5 (CI 85.7–91.4); (iii) women with CHD and women without CVD, and the presence of CVD as factor (CHD women vs non-CVD women model: AUC of 89.4 (CI 85.0–93.9). The 20 bacterial taxa most important identified by each model are shown in Table 4.

**Table 4:** Variable importance of the RF models

Men vs women with CHD model	Importance			CHD men vs non-CVD men model	Importance		CHD women vs non-CVD women model	Importance	
**g_UBA1819 (Ruminococcaceae)**		♀	100.00	o_Peptostreptococcales_Tissierellales	♀	100.00	f_Coriobacteriaceae	♂	100.00
g_Ruminococcus			83.28	**g_Blautia**		89.11	c_Negativicutes	♂	80.17
**g_Bilophila**		♀	67.04	c_Negativicutes	♀	88.56	o_Peptostreptococcales_Tissierellales	♂	77.78
g_Coprobacter			50.15	f_Coriobacteriaceae	♀	80.76	**f_UCG_010 (Oscillospirales)**		73.80
g_Barnesiella			44.85	f_Streptococcaceae	♀	76.89	**g_UCG_010**		68.48
g_Alistipes			43.62	g_Collinsella	♀	68.36	g_Collinsella	♂	58.08
**g_Phascolarctobacterium**		♀	41.33	o_Lactobacillales	♀	67.19	**Ruminococcaceae incertae sedis**		57.79
g_Paraprevotella			38.80	g_Streptococcus	♀	60.19	g_Ruminococcus	♂	41.54
f_Ruminococcaceae			37.38	g_Ruminococcus	♀	57.57	c_Coriobacteriia	♂	37.40
**f_Barnesiellaceae**	♂		37.28	**g_Subdoligranulum**		54.89	f_Streptococcaceae	♂	37.17
g_Eubacterium_siraeum_group			36.53	**c_Bacteroidia**		54.28	**o_Coriobacteriales**		35.78
**g_Subdoligranulum**	♂		36.22	f_Sutterellaceae	♀	53.39	o_Lactobacillales	♂	35.33
g_Parabacteroides			34.08	**g_UCG_002 (Oscillospiraceae)**		48.34	**p_Actinobacteriota**		32.72
o_Oscillospirales			33.61	o_Burkholderiales	♀	47.15	g_Streptococcus	♂	31.54
f_Anaerovoracaceae			32.19	**f_Oscillospiraceae**		45.45	o_Burkholderiales	♂	28.56
f_Tannerellaceae			31.97	**p_Bacteroidota**		44.97	**g_UBA1819 (Ruminococcaceae)**		28.23
f_Prevotellaceae			31.86	**p_Firmicutes**		44.59	**f_Butyricicoccaceae**		27.22
g_Butyricimonas			31.73	**o_Bacteroidales**		42.07	**g_Bilophila**		27.11
**Ruminococcaceae incertae sedis**		♀	31.22	**f_Barnesiellaceae**		41.18	**g_Phascolarctobacterium**		26.92
f_Rikenellaceae			31.17	c_Coriobacteriia	♀	40.66	f_Sutterellaceae	♂	26.08

The sex-specific alterations in gut microbiota related to CHD were selected according to the following criteria: (1) bacterial taxa critical to distinguishing CHD men from non-CVD men or bacterial taxa critical to distinguishing CHD women from non-CVD women (2) bacterial taxa that significantly differentiated CHD men and CHD women. Men vs Women with CHD model: ♂, bacterial taxa that significantly differentiated CHD men and CHD women, which was also present among the main important variables on the CHD men vs non-CVD men model (in bold). ♂ bacterial taxa that significantly differentiated CHD men and CHD women, which was also present among the main important variables on the model CHD women vs non-CVD women model (in bold). CHD men vs non-CVD men model: bold, specific taxa in men model (these taxa did not appear in the CHD women vs non-CVD women model, and they are therefore specific to distinguishing CHD men from non-CVD men). ♂, taxa also present among the main important variables on the CHD women vs non-CVD women model. CHD women vs non-CVD women model: bold, specific taxa in women model (these taxa did not appear in the CHD men vs non-CVD men model, and they are therefore specific to distinguishing CHD women from non-CVD women). ♂, taxa also present among the main important variables on the CHD men vs non-CVD men model

Seven discriminant bacterial taxa, g_UBA1819 (Ruminococcaceae), g_Bilophila, g_Subdoligranulum, g_Phascolarctobacterium, f_Barnesiellaceae, g_Ruminococcus, and Ruminococcaceae incertae sedis, were identified as crucial in distinguishing between CHD men and women, which were also significant in distinguishing between CHD and non-CVD subjects for at least one of the sexes. However, g_Ruminococcus was especially significant in distinguishing between CHD and non-CVD individuals, in both sexes (Table 5).

**Table 5:** Sex-specific alterations in gut microbiota related to CHD

Men vs women with CHD RF model	CHD patients vs non-CVD individuals in RF models for men and women separately
g_UBA1819 (Ruminococcaceae)	Bacterial taxa identified by RF to distinguishing CHD women from non-CVD women
g_Bilophila	Bacterial taxa identified by RF to distinguishing CHD women from non-CVD women
g_Phascolarctobacterium	Bacterial taxa identified by RF to distinguishing CHD women from non-CVD women
f_Barnesiellaceae	Bacterial taxa identified by RF to distinguishing CHD men from non-CVD men
g_Subdoligranulum	Bacterial taxa identified by RF to distinguishing CHD men from non-CVD men
Ruminococcaceae incertae sedis	Bacterial taxa identified by RF to distinguishing CHD women from non-CVD women

The sex-specific alterations in gut microbiota related to CHD were selected according to the following criteria: (1) bacterial taxa critical to distinguishing CHD men from non-CVD men or bacterial taxa critical to distinguishing CHD women from non-CVD women (CHD patients vs non-CVD individuals in models for men and women separately). (2) Bacterial taxa that significantly differentiated CHD men and CHD women (men vs women with CHD model column). *CHD* coronary heart disease, *CVD* cardiovascular disease, *RF* random forest

### Gut microbiota functionality in coronary heart disease patients according to the sex

PICRUSt2 analysis of 16S sequences was used to study the potential function of gut microbiota. Further, STAMP software was employed to impute MetaCyc pathway abundance from the original taxonomic assignment. Data were compared by two-sided Welch’s *t*-test and filtered for false discoveries using the Benjamini–Hochberg method (*Q*-value filter > 0.1) and an effect size filter higher than 0.01 as difference between proportions. Our analysis revealed differences in the abundance of 196 MetaCyc pathways between non-CVD men and non-CVD women, after correcting for multiple comparisons *P*-values < 0.05 and *Q*-values < 0.1. By contrast, we observed differences in the abundance of 18 MetaCyc pathways between CHD men and CHD women, after correcting for multiple comparisons *P*-values < 0.05 and *Q*-values < 0.1 (Additional file 1: Fig. S1). MetaCyc pathways differentially abundant between groups are shown in Additional file 2: Table S1.

## Discussion

This study identifies distinctions in gut bacterial composition between men and women diagnosed with coronary heart disease (CHD). Not only do these differences appear in bacterial composition, but they also exist in terms of beta diversity. Our data modeling technique using random forests (RF) helped us pinpoint significant discriminant bacterial taxa between men and women with CHD. Furthermore, when comparing CHD patients to non-CVD individuals, we noted that some microbial alterations associated with CHD differ between men and women.

Numerous studies underscore the influential role of gut microbiota in CVD development [2, 3]. Such alterations, often termed dysbiosis, include imbalances in bacterial taxa abundance and a reduced microbial diversity linked to CVD [26]—observations also made in our study when comparing CHD patients with non-CVD individuals. Of note, both CHD patients with non-CVD individuals were recruited from the same geographical location and share many of the co-founding factors such as lifestyle, dietary habits and genetic background.

The gut microbiota composition varies with sex [6, 7, 24], which suggests these differences may account for the sex-based discrepancies in CVD incidence. Indeed, several studies propose that such sex-differences could explain the sexual dimorphism observed in autoimmune and metabolic diseases [9–13].

In our study, we noted sex-specific changes in gut microbiota associated with CVD, along with other alterations linked to CVD, but independent of the sex. We utilized a linear discriminant analysis effect size to identify the most discriminant sex-specific alterations in gut microbiota related to CHD. Furthermore, our RF data modeling approach helped identify bacterial taxa that significantly differentiated men from women with CHD and also highlighted taxa critical to distinguishing CHD from non-CVD in sex-segregated models. In addition, the analysis of metagenome prediction of the gut microbiota functionality showed a reduction in number of MetaCyc pathways differently represented between sexes in CHD patients as compared with the number of pathways differently represented between sexes in non-CVD individuals. These results are in line with the reduced microbial diversity linked to CVD [26], also observed in our study; presumably in CHD patients, sex-differences in functionality are also reduced as the diversity in term of functionality is lower in CVD patients than in non-CVD individuals.

Specific alterations in men's intestinal microbiota connected with CHD included a decrease in the abundance of the Barnesiellaceae family, a bacterial taxon associated with CVD [27, 28], and its abundance has been shown to negatively correlate with carotid–femoral pulse wave velocity, a measure of arterial stiffness [29]. Also, we observed an increase in the Subdoligranulum genus, a taxon thought to be beneficial [30], yet its use as a probiotic has failed to show any beneficial effects in preclinical models [31].

On the other hand, alterations in women's intestinal microbiota linked to CHD involved an increase in the abundance of UBA1819 (Ruminococcaceae), Bilophila, and Phascolarctobacterium genera, along with an unknown genus from the Ruminococcaceae family (Ruminococcaceae incertae sedis). The UBA1819 (Ruminococcaceae) genus is connected to rheumatoid arthritis [32, 33], a chronic inflammatory disease like CVD [34], and negatively associated with lactulose and mannitol ratio, an indicator of intestinal barrier dysfunction [35]. A higher abundance in women with CHD compared to men suggests a lower intestinal barrier dysfunction, which may help reduce the CVD incidence in women. *Bilophila* is a sulfite-reducing and hydrogen sulfide-producing genus. This latter triggers direct inflammation, exerts genotoxic and cytotoxic effects on epithelial cells, and impairs gut barrier [36], and therefore involved in the chronic inflammation related with cardiometabolic diseases [37, 38]. *Phascolarctobacterium* a bacterial taxon that has been positively associated to CVD [28]. Even a preclinical study showed that the abundance of this taxon was associated to changes in cardiac structure and function [39].

However, it is worth noting that not all alterations in the gut microbiota connected to CVD were found to be different between sexes. In addition to sex-specific changes, we discovered modifications in the gut microbiota associated with CHD common to both sexes. These pertained to bacterial taxa such as Streptococcus and Ruminococcus genera or Sutterellaceae and Coriobacteriaceae families, which have previously been associated with CVD [40–46]. Of note, the abundance of this bacterial genus was reduced in CHD men as compared with non-CVD men and it was reduced in CHD women as compared with non-CVD women, and additionally, more abundant in CHD men than CHD women. However, the abundance difference was slightly, not detected by LefSe analysis, but enough to be detected in random forest model between CHD men and CHD women.

Taken together, these observations suggest that the abundance of different bacterial taxa related with CVD are differentially altered according to sex, which somehow may influence the sexual dimorphism in its incidence.

Our study has the limitation of an unbalanced number of men and women. In fact, this population was included in the CORDIOPREV study without any type of selection, therefore representing the sexual dimorphism existent in CHD, and any attempt to balance the number of men and women may introduce a bias. In addition, the differences observed herein regarding microbiota architecture may stem from the actual differences in sex hormone levels in elder men and women. On the other hand, it might reflect the residual influence of the dramatic differences in sex steroid profiles early in life between sexes, which may have a persistent effect on gut microbiota over time.

## Perspectives and significance

In summary, our findings suggest that the dysbiosis of the gut microbiota associated with CHD may have sex-specific elements, which could potentially affect the sex-based differences in its incidence. It is of paramount importance to understand the mechanisms behind this sexual dimorphism in the incidence of metabolic and cardiovascular diseases as this could guide the development of effective strategies and therapies aimed at reducing their prevalence and recurrence. Indeed, our results imply that strategies and therapies designed to address gut microbiota dysbiosis should consider sex-specific implications.

### Supplementary Information

**Additional file 1: Figure S1**. Functional characterization between CHD patients according to the sex based on PICRUSt2 analysis. CHD: coronary heart disease patients. Bar chart showing the functional difference between CHD men and CHD women. Data were compared by two-sided Welch’s t-test and filtered for false discoveries using the Benjamini–Hochberg method by using the STAMP software.

**Additional file 2: Table S1**. Functional characterization. Correspondence between BioCyc ID and MetaCyc Pathway nomenclature.

## Data Availability

Collaborations with the Cordioprev Study are open to Biomedical Institutions, always after an accepted proposal for scientific work. Depending on the nature of the collaboration, electronic data, hard copy data, or biological samples should be provided. All collaborations will be made after a collaboration agreement. Terms of the collaboration agreement will be specific for each collaboration, and the extent of the shared documentation (i.e., deidentified participant data, data dictionary, biological samples, hard copy, or other specified data sets) will be also specifically set on the light of each work.
