# Supplementary material for: Sex-specific differences in intestinal microbiota associated with cardiovascular diseases
Source: Biol Sex Differ. 2024 Jan 19;15:7. doi: 10.1186/s13293-024-00582-7 (PMC10797902; doi:10.1186/s13293-024-00582-7)
Supplement: Supplementary file 1 — Additional file 1: Figure S1. Functional characterization between CHD patients according to the sex based on PICRUSt2 analysis. CHD: coronary heart disease patients. Bar chart showing the functional difference between CHD men and CHD women. Data were compared by two-sided Welch’s t-test and filtered for false discoveries using the Benjamini–Hochberg method by using the STAMP software. [file 13293_2024_582_MOESM1_ESM.pdf]

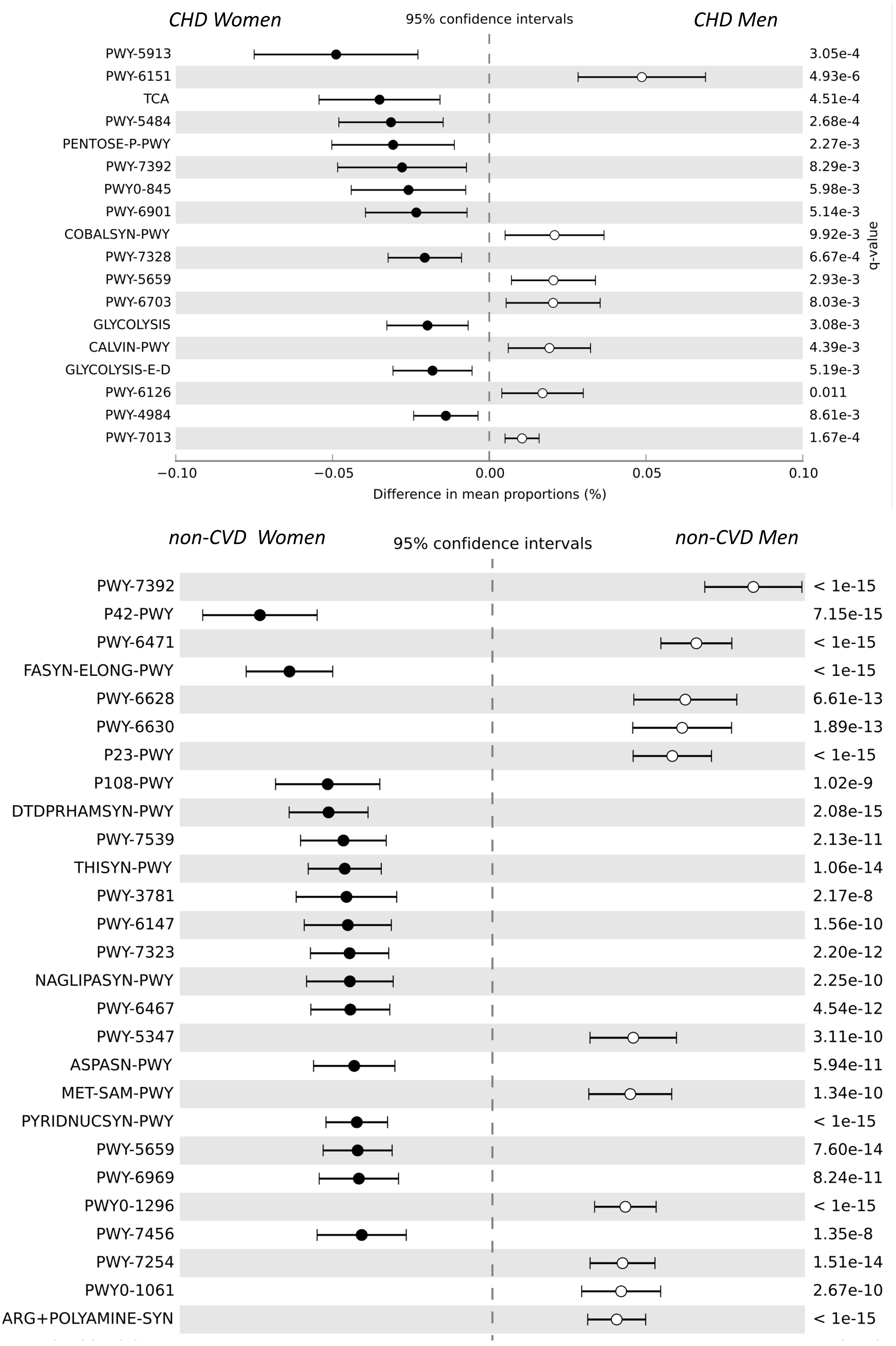

CHD Women
95% confidence intervals
CHD Men
PWY-5913
PWY-6151
TCA
PWY-5484
PENTOSE-P-PWY
PWY-7392
PWY0-845
PWY-6901
COBALSYN-PWY
PWY-7328
PWY-5659
PWY-6703
GLYCOLYSIS
CALVIN-PWY
GLYCOLYSIS-E-D
PWY-6126
PWY-4984
PWY-7013
3.05e-4
4.93e-6
4.51e-4
2.68e-4
2.27e-3
8.29e-3
5.98e-3
5.14e-3
9.92e-3
6.67e-4
2.93e-3
8.03e-3
3.08e-3
4.39e-3
5.19e-3
0.011
8.61e-3
1.67e-4
q-value
−0.10
−0.05
0.00
0.05
0.10
Difference in mean proportions (%)
non-CVD Women
95% confidence intervals
non-CVD Men
PWY-7392
P42-PWY
PWY-6471
FASYN-ELONG-PWY
PWY-6628
PWY-6630
P23-PWY
P108-PWY
DTDPRHAMSYN-PWY
PWY-7539
THISYN-PWY
PWY-3781
PWY-6147
PWY-7323
NAGLIPASYN-PWY
PWY-6467
PWY-5347
ASPASN-PWY
MET-SAM-PWY
PYRIDNUCSYN-PWY
PWY-5659
PWY-6969
PWY0-1296
PWY-7456
PWY-7254
PWY0-1061
ARG+POLYAMINE-SYN
< 1e-15
7.15e-15
< 1e-15
< 1e-15
6.61e-13
1.89e-13
< 1e-15
1.02e-9
2.08e-15
2.13e-11
1.06e-14
2.17e-8
1.56e-10
2.20e-12
2.25e-10
4.54e-12
3.11e-10
5.94e-11
1.34e-10
< 1e-15
7.60e-14
8.24e-11
< 1e-15
1.35e-8
1.51e-14
2.67e-10
< 1e-15

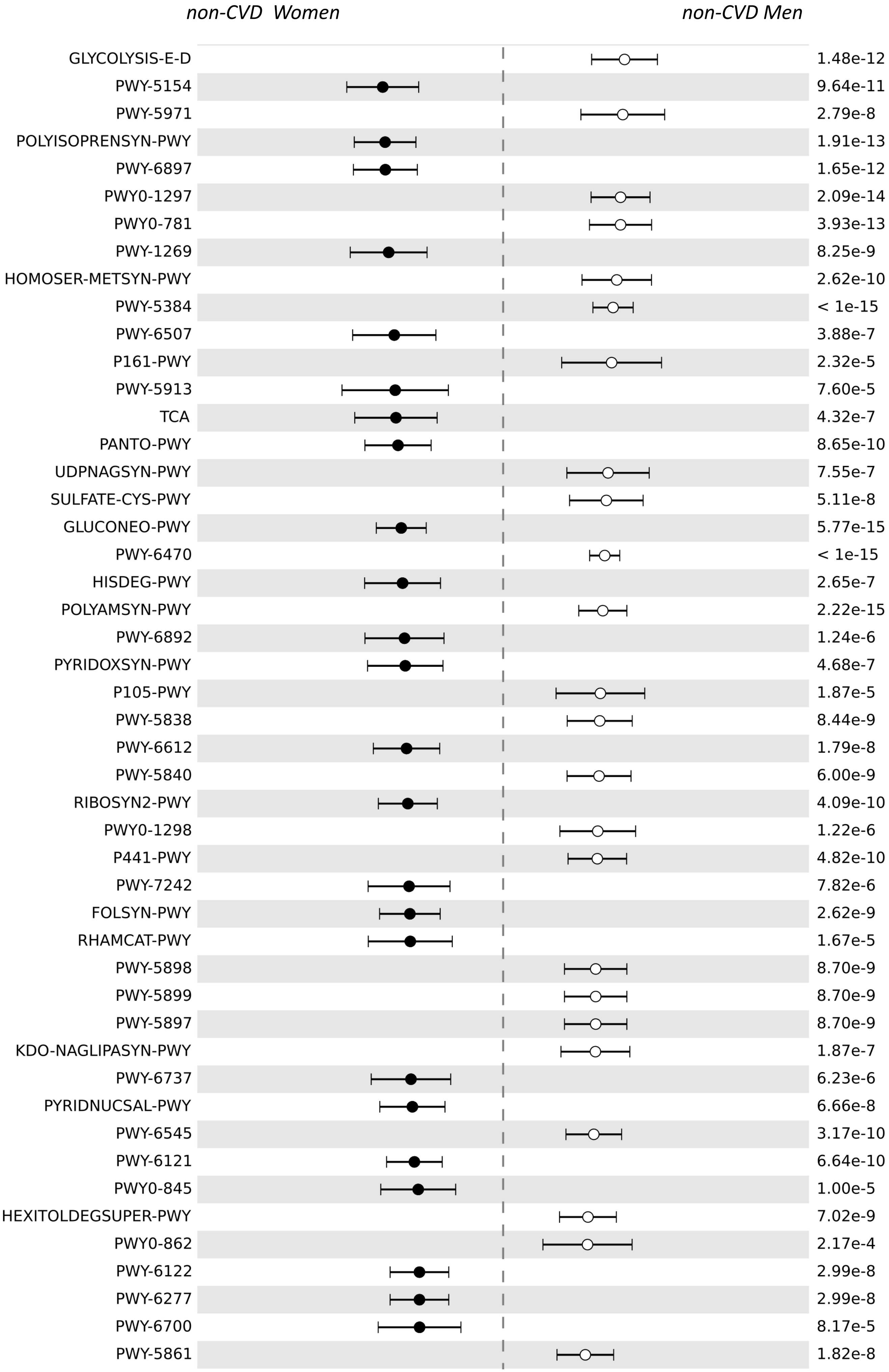

non-CVD Women
non-CVD Men
GLYCOLYSIS-E-D 1.48e-12
PWY-5154 9.64e-11
PWY-5971 2.79e-8
POLYISOPRENSYN-PWY 1.91e-13
PWY-6897 1.65e-12
PWY0-1297 2.09e-14
PWY0-781 3.93e-13
PWY-1269 8.25e-9
HOMOSER-METSYN-PWY 2.62e-10
PWY-5384 < 1e-15
PWY-6507 3.88e-7
P161-PWY 2.32e-5
PWY-5913 7.60e-5
TCA 4.32e-7
PANTO-PWY 8.65e-10
UDPNAGSYN-PWY 7.55e-7
SULFATE-CYS-PWY 5.11e-8
GLUCONEO-PWY 5.77e-15
PWY-6470 < 1e-15
HISDEG-PWY 2.65e-7
POLYAMSYN-PWY 2.22e-15
PWY-6892 1.24e-6
PYRIDOXSYN-PWY 4.68e-7
P105-PWY 1.87e-5
PWY-5838 8.44e-9
PWY-6612 1.79e-8
PWY-5840 6.00e-9
RIBOSYN2-PWY 4.09e-10
PWY0-1298 1.22e-6
P441-PWY 4.82e-10
PWY-7242 7.82e-6
FOLSYN-PWY 2.62e-9
RHAMCAT-PWY 1.67e-5
PWY-5898 8.70e-9
PWY-5899 8.70e-9
PWY-5897 8.70e-9
KDO-NAGLIPASYN-PWY 1.87e-7
PWY-6737 6.23e-6
PYRIDNUCSAL-PWY 6.66e-8
PWY-6545 3.17e-10
PWY-6121 6.64e-10
PWY0-845 1.00e-5
HEXITOLDEGSUPER-PWY 7.02e-9
PWY0-862 2.17e-4
PWY-6122 2.99e-8
PWY-6277 2.99e-8
PWY-6700 8.17e-5
PWY-5861 1.82e-8

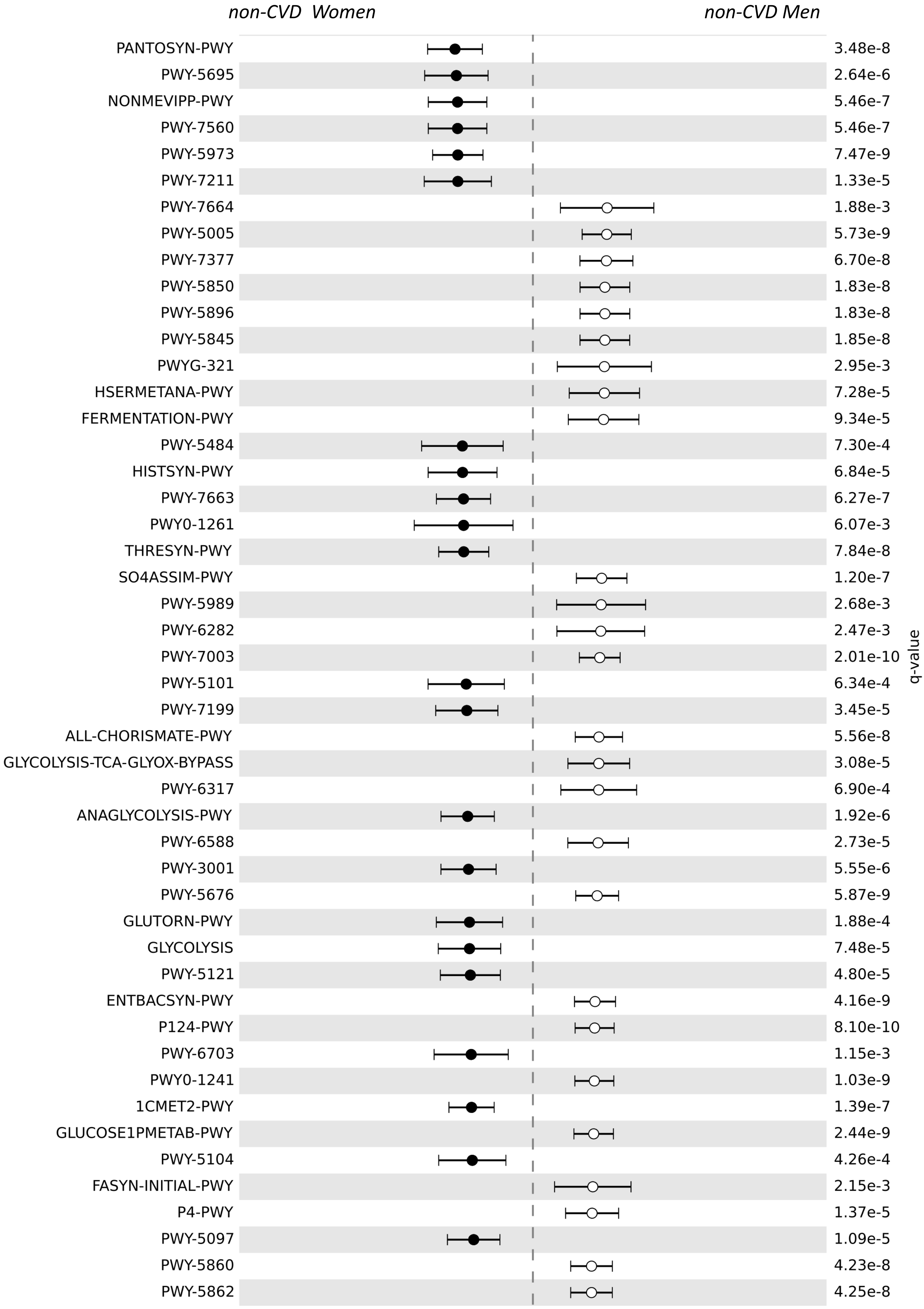
non-CVD Women
non-CVD Men
PANTOSYN-PWY
PWY-5695
NONMEVIPP-PWY
PWY-7560
PWY-5973
PWY-7211
PWY-7664
PWY-5005
PWY-7377
PWY-5850
PWY-5896
PWY-5845
PWYG-321
HSERMETANA-PWY
FERMENTATION-PWY
PWY-5484
HISTSYN-PWY
PWY-7663
PWY0-1261
THRESYN-PWY
SO4ASSIM-PWY
PWY-5989
PWY-6282
PWY-7003
PWY-5101
PWY-7199
ALL-CHORISMATE-PWY
GLYCOLYSIS-TCA-GLYOX-BYPASS
PWY-6317
ANAGLYCOLYSIS-PWY
PWY-6588
PWY-3001
PWY-5676
GLUTORN-PWY
GLYCOLYSIS
PWY-5121
ENTBACSYN-PWY
P124-PWY
PWY-6703
PWY0-1241
1CMET2-PWY
GLUCOSE1PMETAB-PWY
PWY-5104
FASYN-INITIAL-PWY
P4-PWY
PWY-5097
PWY-5860
PWY-5862
3.48e-8
2.64e-6
5.46e-7
5.46e-7
7.47e-9
1.33e-5
1.88e-3
5.73e-9
6.70e-8
1.83e-8
1.83e-8
1.85e-8
2.95e-3
7.28e-5
9.34e-5
7.30e-4
6.84e-5
6.27e-7
6.07e-3
7.84e-8
1.20e-7
2.68e-3
2.47e-3
2.01e-10
6.34e-4
3.45e-5
5.56e-8
3.08e-5
6.90e-4
1.92e-6
2.73e-5
5.55e-6
5.87e-9
1.88e-4
7.48e-5
4.80e-5
4.16e-9
8.10e-10
1.15e-3
1.03e-9
1.39e-7
2.44e-9
4.26e-4
2.15e-3
1.37e-5
1.09e-5
4.23e-8
4.25e-8
q-value

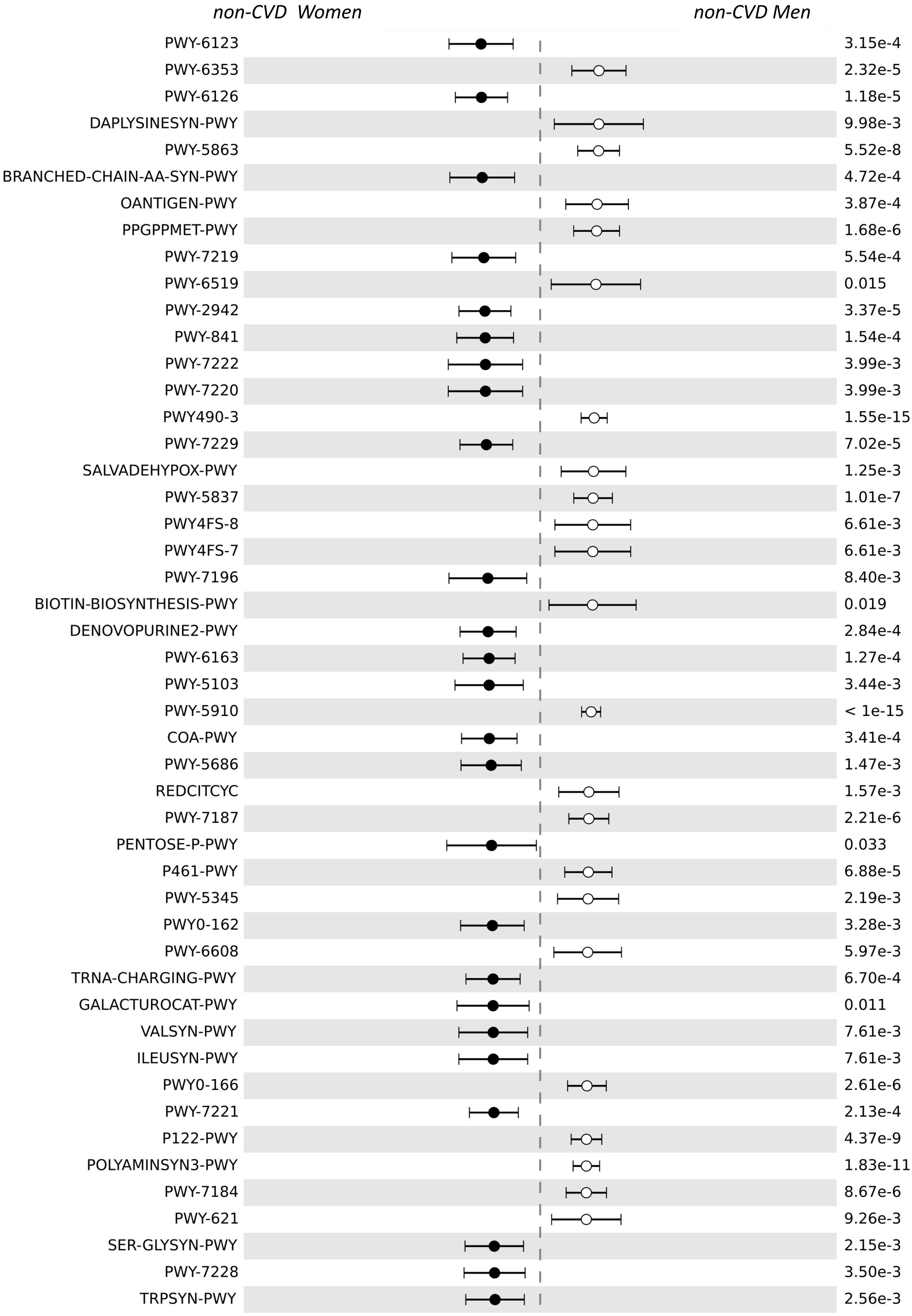
non-CVD Women
non-CVD Men
PWY-6123 3.15e-4
PWY-6353 2.32e-5
PWY-6126 1.18e-5
DAPLYSINESYN-PWY 9.98e-3
PWY-5863 5.52e-8
BRANCHED-CHAIN-AA-SYN-PWY 4.72e-4
OANTIGEN-PWY 3.87e-4
PPGPPMET-PWY 1.68e-6
PWY-7219 5.54e-4
PWY-6519 0.015
PWY-2942 3.37e-5
PWY-841 1.54e-4
PWY-7222 3.99e-3
PWY-7220 3.99e-3
PWY490-3 1.55e-15
PWY-7229 7.02e-5
SALVADEHYPOX-PWY 1.25e-3
PWY-5837 1.01e-7
PWY4FS-8 6.61e-3
PWY4FS-7 6.61e-3
PWY-7196 8.40e-3
BIOTIN-BIOSYNTHESIS-PWY 0.019
DENOVOPURINE2-PWY 2.84e-4
PWY-6163 1.27e-4
PWY-5103 3.44e-3
PWY-5910 < 1e-15
COA-PWY 3.41e-4
PWY-5686 1.47e-3
REDCITCYC 1.57e-3
PWY-7187 2.21e-6
PENTOSE-P-PWY 0.033
P461-PWY 6.88e-5
PWY-5345 2.19e-3
PWY0-162 3.28e-3
PWY-6608 5.97e-3
TRNA-CHARGING-PWY 6.70e-4
GALACTUROCAT-PWY 0.011
VALSYN-PWY 7.61e-3
ILEUSYN-PWY 7.61e-3
PWY0-166 2.61e-6
PWY-7221 2.13e-4
P122-PWY 4.37e-9
POLYAMINSYN3-PWY 1.83e-11
PWY-7184 8.67e-6
PWY-621 9.26e-3
SER-GLYSYN-PWY 2.15e-3
PWY-7228 3.50e-3
TRPSYN-PWY 2.56e-3

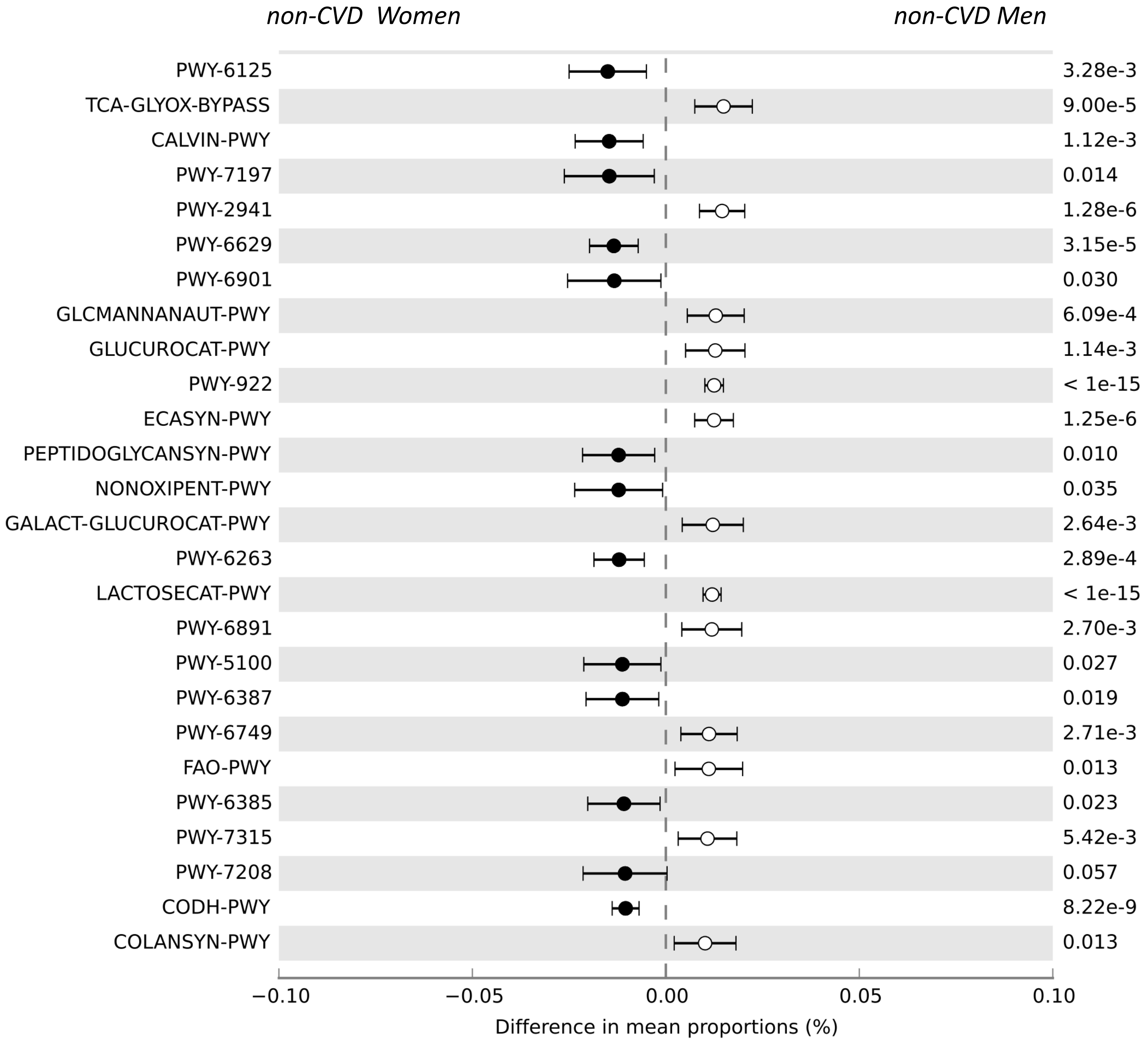

Figure S1. Functional characterization between CHD patients according to the sex based on PICRUSt2 analysis.
