# Supplementary material for: Sex-specific differences in intestinal microbiota associated with cardiovascular diseases
Source: Biol Sex Differ. 2024 Jan 19;15:7. doi: 10.1186/s13293-024-00582-7 (PMC10797902; doi:10.1186/s13293-024-00582-7)
Supplement: Supplementary file 2 — Additional file 2: Table S1. Functional characterization. Correspondence between BioCyc ID and MetaCyc Pathway nomenclature. [file 13293_2024_582_MOESM2_ESM.pdf]

CHD men versus CHD women

BioCyc ID	***MetaCyc Pathway***
PWY-5913	partial TCA cycle (obligate autotrophs)
PWY-6151	S-adenosyl-L-methionine salvage I
TCA	TCA
PWY-5484	glycolysis II (from fructose 6-phosphate)
PENTOSE-P-PWY	pentose phosphate pathway
PWY-7392	taxadiene biosynthesis (engineered)
PWY0-845	superpathway of pyridoxal 5'-phosphate biosynthesis and salvage
PWY-6901	superpathway of glucose and xylose degradation
COBALSYN-PWY	superpathway of adenosylcobalamin salvage from cobinamide I
PWY-7328	superpathway of UDP-glucose-derived O-antigen building blocks biosynthesis
PWY-5659	GDP-mannose biosynthesis
PWY-6703	preQ0 biosynthesis
GLYCOLYSIS	GLYCOLYSIS
CALVIN-PWY	Calvin-Benson-Bassham cycle
GLYCOLYSIS-E-D	superpathway of glycolysis and the Entner-Doudoroff pathway
PWY-6126	superpathway of adenosine nucleotides de novo biosynthesis II
PWY-4984	urea cycle
PWY-7013	(S)-propane-1,2-diol degradation

non-CVD men versus non-CVD women

BioCyc ID	***MetaCyc Pathway***
PWY-7392	taxadiene biosynthesis (engineered)
P42-PWY	incomplete reductive TCA cycle
PWY-6471	peptidoglycan biosynthesis IV(Enterococcus faecium)
FASYN-ELONG-PWY	fatty acid elongation -- saturated
PWY-6628	superpathway of L-phenylalanine biosynthesis
PWY-6630	superpathway of L-tyrosine biosynthesis
P23-PWY	reductive TCA cycle I
P108-PWY	pyruvate fermentation to propanoate I
DTDPRHAMSYN-PWY	dTDP-β-L-rhamnose biosynthesis
PWY-7539	6-hydroxymethyl-dihydropterin diphosphate biosynthesis III (Chlamydia)
THISYN-PWY	superpathway of thiamine diphosphate biosynthesis I
PWY-3781	aerobic respiration I (cytochrome c)
PWY-6147	hydroxymethyl-dihydropterin diphosphate biosynthesis I
PWY-7323	superpathway of GDP-mannose-derived O-antigen building blocks biosynthesis
NAGLIPASYN-PWY	lipid IVA biosynthesis (E. coli)
PWY-6467	Kdo transfer to lipid IVA (Chlamydia)
PWY-5347	superpathway of L-methionine biosynthesis (transsulfuration)
ASPASN-PWY	superpathway of L-aspartate and L-asparagine biosynthesis
MET-SAM-PWY	superpathway of S-adenosyl-L-methionine biosynthesis

PYRIDNUCSYN-PWY	NAD de novo biosynthesis I
PWY-5659	GDP-mannose biosynthesis
PWY-6969	TCA cycle V (2-oxoglutarate synthase)
PWY0-1296	purine ribonucleosides degradation
PWY-7456	β-(1,4)-mannan degradation
PWY-7254	TCA cycle VII (acetate-producers)
PWY0-1061	superpathway of L-alanine biosynthesis
ARG+POLYAMINE-SYN	superpathway of arginine and polyamine biosynthesis
GLYCOLYSIS-E-D	superpathway of glycolysis and the Entner-Doudoroff pathway
PWY-5154	L-arginine biosynthesis III (via N-acetyl-L-citrulline)
PWY-5971	palmitate biosynthesis II (type II fatty acid synthase)
POLYISOPRENSYN-PWY	polyisoprenoid biosynthesis (E. coli)
PWY-6897	thiamine diphosphate salvage II
PWY0-1297	superpathway of purine deoxyribonucleosides degradation
PWY0-781	aspartate superpathway
PWY-1269	CMP-3-deoxy-D-manno-octulosonate biosynthesis
HOMOSER-METSYN-PWY	L-methionine biosynthesis I
PWY-5384	sucrose degradation IV (sucrose phosphorylase)
PWY-6507	4-deoxy-L-threo-hex-4-enopyranuronate degradation
P161-PWY	acetylene degradation (anaerobic)
PWY-5913	partial TCA cycle (obligate autotrophs)
TCA	TCA cycle I (prokaryotic)
PANTO-PWY	phosphopantothenate biosynthesis I
UDPNAGSYN-PWY	UDP-N-acetyl-D-glucosamine biosynthesis I
SULFATE-CYS-PWY	superpathway of sulfate assimilation and cysteine biosynthesis
GLUCONEO-PWY	gluconeogenesis I
PWY-6470	peptidoglycan biosynthesis V (β-lactam resistance)
HISDEG-PWY	L-histidine degradation I
POLYAMSYN-PWY	superpathway of polyamine biosynthesis I
PWY-6892	thiazole component of thiamine diphosphate biosynthesis I
PYRIDOXSYN-PWY	pyridoxal 5'-phosphate biosynthesis I
P105-PWY	TCA cycle IV (2-oxoglutarate decarboxylase)
PWY-5838	superpathway of menaquinol-8 biosynthesis I
PWY-6612	superpathway of tetrahydrofolate biosynthesis
PWY-5840	superpathway of menaquinol-7 biosynthesis
RIBOSYN2-PWY	flavin biosynthesis I (bacteria and plants)
PWY0-1298	superpathway of pyrimidine deoxyribonucleosides degradation
P441-PWY	superpathway of N-acetylneuraminate degradation
PWY-7242	D-fructuronate degradation
FOLSYN-PWY	superpathway of tetrahydrofolate biosynthesis and salvage
RHAMCAT-PWY	L-rhamnose degradation I
PWY-5898	superpathway of menaquinol-12 biosynthesis
PWY-5899	superpathway of menaquinol-13 biosynthesis

PWY-5897	superpathway of menaquinol-11 biosynthesis
KDO-NAGLIPASYN-PWY	superpathway of (Kdo)2-lipid A biosynthesis
PWY-6737	starch degradation V
PYRIDNUCSAL-PWY	NAD salvage pathway I (PNC VI cycle)
PWY-6545	pyrimidine deoxyribonucleotides de novo biosynthesis III
PWY-6121	5-aminoimidazole ribonucleotide biosynthesis I
PWY0-845	superpathway of pyridoxal 5'-phosphate biosynthesis and salvage
HEXITOLDEGSUPER-PWY	superpathway of hexitol degradation (bacteria)
PWY0-862	(5Z)-dodecenoate biosynthesis I
PWY-6122	5-aminoimidazole ribonucleotide biosynthesis II
PWY-6277	superpathway of 5-aminoimidazole ribonucleotide biosynthesis
PWY-6700	queuosine biosynthesis I (de novo)
PWY-5861	superpathway of demethylmenaquinol-8 biosynthesis I
PANTOSYN-PWY	superpathway of coenzyme A biosynthesis I (bacteria)
PWY-5695	inosine 5'-phosphate degradation
NONMEVIPP-PWY	methylerythritol phosphate pathway I
PWY-7560	methylerythritol phosphate pathway II
PWY-5973	cis-vaccenate biosynthesis
PWY-7211	superpathway of pyrimidine deoxyribonucleotides de novo biosynthesis
PWY-7664	oleate biosynthesis IV (anaerobic)
PWY-5005	biotin biosynthesis II
PWY-7377	cob(II)yrinate a,c-diamide biosynthesis I (early cobalt insertion)
PWY-5850	superpathway of menaquinol-6 biosynthesis
PWY-5896	superpathway of menaquinol-10 biosynthesis
PWY-5845	superpathway of menaquinol-9 biosynthesis
PWYG-321	mycolate biosynthesis
HSERMETANA-PWY	L-methionine biosynthesis III
FERMENTATION-PWY	mixed acid fermentation
PWY-5484	glycolysis II (from fructose 6-phosphate)
HISTSYN-PWY	L-histidine biosynthesis
PWY-7663	gondoate biosynthesis (anaerobic)
PWY0-1261	peptidoglycan recycling I
THRESYN-PWY	superpathway of L-threonine biosynthesis
SO4ASSIM-PWY	assimilatory sulfate reduction I
PWY-5989	stearate biosynthesis II (bacteria and plants)
PWY-6282	palmitoleate biosynthesis I (from (5Z)-dodec-5-enoate)
PWY-7003	lycerol degradation to butanol
PWY-5101	L-isoleucine biosynthesis II
PWY-7199	pyrimidine deoxyribonucleosides salvage
ALL-CHORISMATE-PWY	superpathway of chorismate metabolism
GLYCOLYSIS-TCA-GLYOX-BYPASS	superpathway of glycolysis, pyruvate dehydrogenase, TCA, and glyoxylate bypass
PWY-6317	D-galactose degradation I (Leloir pathway)
ANAGLYCOLYSIS-PWY	glycolysis III (from glucose)

PWY-6588	pyruvate fermentation to acetone
PWY-3001	superpathway of L-isoleucine biosynthesis I
PWY-5676	acetyl-CoA fermentation to butanoate
GLUTORN-PWY	L-ornithine biosynthesis I
GLYCOLYSIS	glycolysis I (from glucose 6-phosphate)
PWY-5121	superpathway of geranylgeranyl diphosphate biosynthesis II (via MEP)
ENTBACSYN-PWY	enterobactin biosynthesis
P124-PWY	Bifidobacterium shunt
PWY-6703	preQ0 biosynthesis
PWY0-1241	ADP-L-glycero-β-D-manno-heptose biosynthesis
1CMET2-PWY	folate transformations III (E. coli)
LUCOSE1PMETAB-PWY	glucose and glucose-1-phosphate degradation
PWY-5104	L-isoleucine biosynthesis IV
FASYN-INITIAL-PWY	superpathway of fatty acid biosynthesis initiation
P4-PWY	superpathway of L-lysine, L-threonine and L-methionine biosynthesis I
PWY-5097	L-lysine biosynthesis VI
PWY-5860	superpathway of demethylmenaquinol-6 biosynthesis I
PWY-5862	superpathway of demethylmenaquinol-9 biosynthesis
PWY-6123	inosine-5'-phosphate biosynthesis I
PWY-6126	superpathway of adenosine nucleotides de novo biosynthesis II
DAPLYSINESYN-PWY	L-lysine biosynthesis I
PWY-5863	superpathway of phylloquinol biosynthesis
BRANCHED-CHAIN-AA-SYN-PWY	superpathway of branched chain amino acid biosynthesis
OANTIGEN-PWY	O-antigen building blocks biosynthesis (E. coli)
PPGPPMET-PWY	ppGpp metabolism
PWY-7219	adenosine ribonucleotides de novo biosynthesis
PWY-6519	8-amino-7-oxononanoate biosynthesis I
PWY-2942	L-lysine biosynthesis III
PWY-841	superpathway of purine nucleotides de novo biosynthesis I
PWY-7222	guanosine deoxyribonucleotides de novo biosynthesis II
PWY-7220	adenosine deoxyribonucleotides de novo biosynthesis II
PWY490-3	nitrate reduction VI (assimilatory)
PWY-7229	superpathway of adenosine nucleotides de novo biosynthesis I
SALVADEHYPOX-PWY	adenosine nucleotides degradation II
PWY-5837	2-carboxy-1,4-naphthoquinol biosynthesis
PWY4FS-8	phosphatidylglycerol biosynthesis II
PWY4FS-7	phosphatidylglycerol biosynthesis I
PWY-7196	superpathway of pyrimidine ribonucleosides salvage
BIOTIN-BIOSYNTHESIS-PWY	biotin biosynthesis I
DENOVOPURINE2-PWY	superpathway of purine nucleotides de novo biosynthesis II
PWY-6163	chorismate biosynthesis from 3-dehydroquinate
PWY-5103	L-isoleucine biosynthesis III
PWY-5910	superpathway of geranylgeranyldiphosphate biosynthesis I (via mevalonate)

COA-PWY	coenzyme A biosynthesis I (bacteria)
PWY-5686	UMP biosynthesis I
REDCITCYC	TCA cycle VI (Helicobacter)
PWY-7187	pyrimidine deoxyribonucleotides de novo biosynthesis II
PENTOSE-P-PWY	pentose phosphate pathway
P461-PWY	hexitol fermentation to lactate, formate, ethanol and acetate
PWY-5345	superpathway of L-methionine biosynthesis (by sulfhydrylation)
PWY0-162	superpathway of pyrimidine ribonucleotides *de novo* biosynthesis
PWY-6608	guanosine nucleotides degradation III
TRNA-CHARGING-PWY	tRNA charging
GALACTUROCAT-PWY	D-galacturonate degradation I
VALSYN-PWY	L-valine biosynthesis
ILEUSYN-PWY	L-isoleucine biosynthesis I (from threonine)
PWY0-166	superpathway of pyrimidine deoxyribonucleotides de novo biosynthesis (E. coli)
PWY-7221	guanosine ribonucleotides de novo biosynthesis
P122-PWY	heterolactic fermentation
POLYAMINSYN3-PWY	superpathway of polyamine biosynthesis II
PWY-7184	pyrimidine deoxyribonucleotides *de novo* biosynthesis I
PWY-621	sucrose degradation III (sucrose invertase)
SER-GLYSYN-PWY	superpathway of L-serine and glycine biosynthesis I
PWY-7228	superpathway of guanosine nucleotides *de novo* biosynthesis I
TRPSYN-PWY	L-tryptophan biosynthesis
PWY-6125	superpathway of guanosine nucleotides *de novo* biosynthesis II
TCA-GLYOX-BYPASS	superpathway of glyoxylate bypass and TCA
CALVIN-PWY	Calvin-Benson-Bassham cycle
PWY-7197	pyrimidine deoxyribonucleotide phosphorylation
PWY-2941	L-lysine biosynthesis II
PWY-6629	superpathway of L-tryptophan biosynthesis
PWY-6901	superpathway of glucose and xylose degradation
GLCMANNANAUT-PWY	superpathway of *N*-acetylglucosamine, *N*-acetylmannosamine and *N*-acetylneuraminate degradation
GLUCUROCAT-PWY	superpathway of β-D-glucuronosides degradation
PWY-922	mevalonate pathway I (eukaryotes and bacteria)
ECASYN-PWY	enterobacterial common antigen biosynthesis
PEPTIDOGLYCANSYN-PWY	peptidoglycan biosynthesis I (*meso*-diaminopimelate containing)
NONOXIPENT-PWY	pentose phosphate pathway (non-oxidative branch) I
GALACT-GLUCUROCAT-PWY	superpathway of hexuronide and hexuronate degradation
PWY-6263	superpathway of menaquinol-8 biosynthesis II
LACTOSECAT-PWY	lactose degradation I
PWY-6891	thiazole component of thiamine diphosphate biosynthesis II
PWY-5100	pyruvate fermentation to acetate and lactate II
PWY-6387	UDP-*N*-acetylmuramoyl-pentapeptide biosynthesis I (*meso*-diaminopimelate containing)
PWY-6749	CMP-legionaminate biosynthesis I

FAO-PWY	fatty acid β-oxidation I (generic)
PWY-6385	peptidoglycan biosynthesis III (mycobacteria)
PWY-7315	dTDP-*N*-acetylthomosamine biosynthesis
PWY-7208	superpathway of pyrimidine nucleobases salvage
CODH-PWY	reductive acetyl coenzyme A pathway I (homoacetogenic bacteria)
COLANSYN-PWY	colanic acid building blocks biosynthesis

Table S1. Functional characterization. Correspondence between BioCyc ID and MetaCyc Pathway nomenclature.
